# Urban Environments, Health, and Environmental Sustainability: Findings From the SALURBAL Study

**DOI:** 10.1007/s11524-024-00932-1

**Published:** 2024-11-25

**Authors:** Ana V. Diez Roux, Marcio Alazraqui, Tania Alfaro, Tonatiuh Barrientos-Gutierrez, Waleska T. Caiaffa, M. Fernanda Kroker-Lobos, J. Jaime Miranda, Daniel Rodriguez, Olga Lucia Sarmiento, Alejandra Vives, SALURBAL Study

**Affiliations:** 1 Drexel Urban Health Collaborative, Dornsife School of Public Health, Drexel University, Philadelphia, PA USA; 2 Instituto de Salud Colectiva, Universidad Nacional de Lanus, Buenos Aires, Argentina; 3 Universidad de Chile, Santiago, Chile; 4 Instituto Nacional de Salud Pública (INSP), Mexico City, Mexico; 5 Universidade Federal de Minas Gerais, Belo Horizonte, Brazil; 6 Instituto de Nutrición de Centroamérica y Panamá, Guatemala City, Guatemala; 7 Sydney School of Public Health, The University of Sydney, Sydney, Australia; 8 Institute for Transportation Studies, University of California at Berkeley, Berkeley, CA USA; 9 Universidad de los Andes, Bogotá, Colombia; 10 Pontificia Universidad Católica de Chile, Santiago, Chile

**Keywords:** Urban health, Health inequities, Policy, Environment, Global health, Epidemiology

## Abstract

Despite the relevance of cities and city policies for health, there has been limited examination of large numbers of cities aimed at characterizing urban health determinants and identifying effective policies. The relatively few comparative studies that exist include few cities in lower and middle income countries. The *Salud Urbana en America Latina* study (SALURBAL) was launched in 2017 to address this gap. The study has four aims: (1) to investigate social and physical environment factors associated with health differences across and within cities; (2) to document the health impact of urban policies and interventions; (3) to use systems approaches to better understand dynamics and identify opportunities for intervention and (4) to create a new dialogue about the drivers of health in cities and their policy implications and support action. Beyond these aims SALURBAL, has an overarching goal of supporting collaborative policy relevant research and capacity -building that engages individuals and institutions from across Latin America. In this review we provide an update on the SALURBAL data resource and collaborative approach and summarize key findings from the first aim of the study. We also describe key elements of our approach, challenges we have faced and how we have overcome them, and identify key opportunities to support policy relevant evidence generation in urban health for the future.

**Supplementary Information:**

The online version contains supplementary material available at 10.1007/s11524-024-00932-1.

## Introduction

Urbanization is increasing worldwide, especially in low- and middle-income countries (LMICs)[1]. Despite the relevance of cities and city policies for health, there has been limited examination of the drivers of health across large numbers of cities. The few comparative studies that exist include few cities in LMIC and do not engage local teams across large regions.

The Salud Urbana en America Latina Study (SALURBAL) was launched in 2017 to address this gap [2]. The study has four aims: (1) to investigate social and physical environment factors associated with health differences across and within cities; (2) to document the health impact of urban policies and interventions; (3) to use systems approaches to better understand dynamics and identify opportunities for intervention and (4) to create a new dialogue about the drivers of health in cities and their policy implications and support action. The three first aims encompass the multiple sources of evidence needed to inform understanding. The fourth aim was designed to maximize stakeholder input and support dissemination activities to scientists, policy makers and the public in ways that promote transformative action.

In this review we summarize the SALURBAL approach to regionally engaged collaborative research and summarize select findings derived from the first aim of the study. We also describe challenges we have faced and identify opportunities for the future.

## Methods

The SALURBAL Project, coordinated by the Drexel University Urban Health Collaborative, has engaged 11 institutions in Latin America based in 7 different countries, with one of them representing 5 countries in Central America, as well as two US partners [2]. An Executive Committee meets regularly to review progress and set directions. A monthly team meeting including all investigators and trainees across all institutions provides regular updates and shares works in progress. The work in support of the first SALURBAL Aim (the focus of this paper) was organized via a series of Cores and Working groups (Table 1). As of 2023 the team included more than 200 members who regularly participate in team meetings and engage in SALURBAL activities, and 102 members who are supported by the study in various ways. The SALURBAL network evolved over time expanding across disciplines, sectors, and geography [3].

**Table 1:** Collaborative groups related to Aim 1 in SALURBAL

**Type**	**Group**	**Objective**
Support Cores	Data and Methods Core	Oversee data compilation and harmonization of health (vital statistics and survey) and census data, linkages to environmental data, create documentation and datasets, support analyses.
	Physical and Built Environment Core	Lead identification, and creation of physical and built environment data, support the development of research questions and analyses using this data.
	Social Environment Core	Lead identification, and creation of social environment data, support the development of research questions and analyses using this data.
Working groups	Maternal and child health	Provide a forum for development of research questions, discussions of findings, and identification of additional collaborative opportunities involving infant mortality, birth rates, and perinatal outcomes.
	COVID-19/infectious diseases	Support compilation and enhancement of infectious disease data, including COVID-19. Develop research questions and discuss findings and analytical approaches.
	Climate change	Provide a forum for exploration of climate data and discussion of climate change questions relevant to SALURBAL.
	Food Environment	Provide a forum for development of research questions, discussions of findings, and identification of additional collaborative opportunities involving diet and food environment. Support harmonization of dietary data.
	Residential segregation and racial inequities	Provide a forum for development of research questions, discussions of findings, and identification of additional collaborative opportunities involving racial inequities and segregation. Support creation of segregation measures.
	Gender	Provide a forum for development of research questions, discussions of findings, and identification of additional collaborative opportunities involving gender inequities.
	Parks and health	Support the creation, analysis and interpretation of measures of access to parks in SALURBAL.
Analysis groups	Life Events	Support and coordinate analyses using vital statistics data (mortality, births) via works in progress presentations and consultation follow up as needed.
	Survey Analysis	Support and coordinate analyses of survey data using via works in progress presentations and consultation follow up as needed.
	Small area analyses	Support estimation and analyses of outcomes for small areas (eg neighborhoods) within cities. Advance papers involving small area analyses.

### A Flexible Data Resource to Support Urban Health Research Across the Region

A first step in the SALURBAL collaborative process was the creation of a data infrastructure that would allow the characterization of cities at various geographic levels, over time, and across multiple domains. The SALURBAL cities encompassed all urban agglomerations of 100,000 people or more in 2010 in 11 participating countries (Argentina, Brazil, Chile, Colombia, Costa Rica, El Salvador, Guatemala, Mexico, Nicaragua, Panama, Peru). SALURBAL cities were identified by combining information from multiple data sources, [4] and operationalized in various standardized ways [4] [5].

Data domains of interest were defined a priori. Cross-country teams oversaw the compilation and harmonization of data using multiple sources and in close collaboration with regional partners (Table 2). Data were linked using a standardized set of geographic definitions, as well as indicators for time (year, month and even day when available). Issues of data quality and limitations in coverage or comparability were highlighted in data documentation. A number of steps were used to improve data [6–9]. Select data challenges and how they were addressed are shown in Table 3.

**Table 2:** Summary of key SALURBAL data compiled for SALURBAL Aim 1

**Domain**	**Level of measurement**	Time frame*	**Sample measures**	**Source**
**Health**				
Mortality and births	Cities, subcities**, neighborhoods (select countries)	Annual, monthly/daily in some cases	Age-adjusted all cause and cause specific rates. Life expectancy at various ages; Birth rates; Prevalence of low birth weight.	Country vital statistics registries
Self-reported health/mental health	Individual-level linked to cities, subcities and neighborhoods (select countries)	Select years	Prevalence of poor health, depressive symptoms scores.	Surveys conducted by country agencies including Ministries of Health, Family Welfare Institutes, and National Statistical Agencies
Health risks factors	Individual-level linked to cities, subcities and neighborhoods (select countries)	Select years	Prevalence of diabetes and hypertension, body mass index; cancer screening rates.	Surveys conducted by country agencies including Ministries of Health, Family Welfare Institutes, and National Statistical Agencies
**Population characteristics**				
Total population by age, gender and race/ethnicity	Cities, subcities, and neighborhoods (some indicators)	Census years and interpolations and projections		National census offices
Social and economic characteristics	Cities, subcities, and neighborhoods (some indicators)	Census years and interpolations	Percent population with high school education; living below poverty; unemployment; Income Gini coefficient; segregation; gender equity; GDP.	Organization for Economic Co-operation and Development (OECD) data, national censuses and household surveys conducted by country national census offices and national statistical agencies.
**Housing and services**	Cities, subcities, and neighborhoods (some indicators)	Census years and interpolations	Percent durable walls and floors; overcrowding; access to water and sanitation.	National censuses.
**Health care**	Provinces/states, cities (some indicators)	Select years	Physicians per capita; hospital beds per capita.	National health ministries and statistical agencies, World Health Organization (WHO).
**Built environment and transportation**				
Urban form and landscape	Cities and subcities	Select years	Population density; Fragmentation; Isolation.	Modelled land surface from the DLR’s Global Urban Footprint (GUF); population from national censuses and Worldpop; greenspaces from a supervised classifier using Sentinel-2 images from 2017; greenness processed from MODIS (MOD13Q1.006).
Streets	Cities, subcities, and neighborhoods (some indicators)	Select year	Street connectivity and density.	Street network from OpenStreetMap
Transportation	Cities and subcities	Select year	Vehicle registration; congestion index; BRT/subways/trams.	Local and national transportation agencies, national statistical agencies, brtdata.org website, road safety agencies, OpenStreetMap, and Google Maps.
Parks	Cities, subcities, and neighborhoods (some indicators)	Select year	Size, number, and density of parks.	Satellite images from Sentinel-2 and Google Cloud collaborators database.
**Air pollution, temperature, and natural environment**				
Air pollution and emissions	Cities, subcities and neighborhoods	Daily, monthly and annual	Particulate matter less than 2.5 microns (PM2.5) and nitrogen dioxide (NO2); Carbon footprint.	Monitor data from any available air quality monitoring systems in each country, modelled satellite data from Atmospheric Composition Analysis Group (ATMOS), and Gridded Global Model of City Footprints (GGMCF).
Green space	Cities, subcities and neighborhoods	Annual, select year	Maximum and median Normalized Difference Vegetation Index (NDVI); Percentage of green space, green space fragmentation and isolation.	Modelled satellite data from NASA Earth Observatory and OpenStreetMap.
Temperature	Cities and subcities	Daily	Mean and maximum daily temperature.	Modelled 2-m air temperature from ERA5-Land available from the European Center for Medium-Range Forecasts (ECMWF) with machine learning imputation to address missing values.

^*^For most measures time span is 2000–2020 but only select time points may be available

^**^Subcities are administrative units within cities such as municipalities or similar

Additional details on sources and variables can be found at data.lacurbanhealth.org

**Table 3:** Key data challenges encountered by SALURBAL and examples of how they were addressed

**Domain**	**Sample challenge**	**How it was addressed**
Data availability	Not all countries have same data availability.	Develop a data system that allows incorporation of whatever data is available, for whatever timeframe or level of aggregation is available. Allow papers that focus on all cities as well as single countries or even single cities to take full advantage of data available. Focus on data (eg, remote sensing, congestion index, parks) that can be collected systematically across countries and develop protocols to do so.
Data access	Access to data differs across country and type or data.	Develop flexible Data Use Agreements that can be adapted as needed. Work with country teams to develop relationships and partnerships with local agencies. Support local needs (eg georeferencing).
Lack of comparability of measures	Measures available (eg in surveys or in censuses) differ across countries	Harmonize using or adapting previously used measures whenever possible. Create several versions or harmonized variables that allow inclusion of different sets of variables.
Sampling differences	Surveys employ various sampling strategies and cover different populations.	Analyze with attention to sampling issues, including adjustment factors as necessary. Develop methods to derive needed estimates when possible.
Missing data or ill-defined categories	Data is missing (eg age in death certificates) or lumped into categories not useful for analysis (eg ill defined causes). Temperature data is missing for some areas.	Use appropriate imputation and readjudication strategies based on prior work whenever possible.
Sparse data	Small numbers of events create instability in estimates	Develop and use methods appropriate for small area analyses.
Lack of environmental data	Air pollution monitors not available in many locations.	Use modelled estimates derived from other sources to systematically assess cities across the sample. Calculate and model our own data (greenness, greenspaces, water) from remote sensing data.
Definition of geographic units changes over time	New municipalities are created and/or boundaries are redefined.	Create cross-walks that identify time invariant geographic units; create “harmonized” definitions by combining various units at different time point; flag units with large changes.
Data collected at different time points for different countries	Temporal coverage varies, creating unevenness in the availability of data over the years.	Develop methods to impute or approximate data for years with missing information; aggregate data across time.

These efforts led to the creation of a unique data resource spanning multiple years, and across various geographic definitions encompassing all 371 cities. This data resource and associated metadata followed FAIR principles of findability, accessibility, interoperability, and reusability[10]. All data that can be made publicly available is made available on the SALURBAL Portal which also contains some data visualizations and various products SALURBAL has created [5].

### A Collaborative Scientific Process: the SALURBAL Approach 

**Fig. 1:**
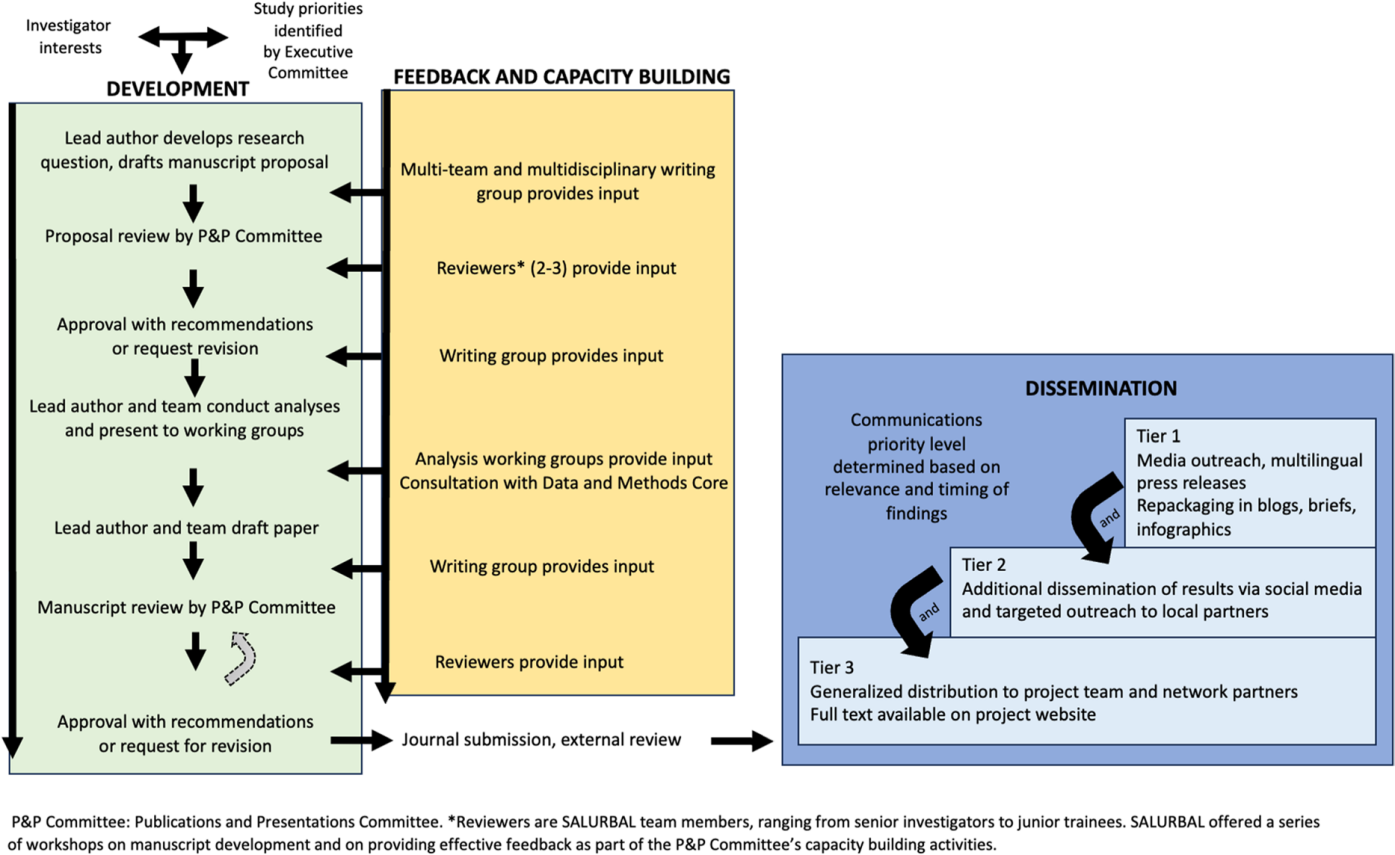
Overview of SALURBAL publication development, review, and dissemination process

To support collaboration while ensuring productivity and quality, SALURBAL created a collaborative process for the production and dissemination of scientific findings (Fig. 1). Key elements included (1) a clear publications and presentations policy with guidelines for authorship and collaboration; (2) internal peer review of all manuscript proposals and final manuscripts coordinated and managed by a Publications and Presentations Committee; (3) creation of working groups around thematic topics to support analyses and interpretation; (4) delivery of analysis workshops and consultations as well as training and support of local analysts guided by a study Data and Methods Core; (5) review of full manuscripts and scientific presentations before submission by SALURBAL reviewers with opportunity for revision; and (6) oversight by the study executive committee to ensure that priority analyses were advancing. A dedicated dissemination team supported communicating findings via briefs, webinars and media outreach [11].

## Results

We summarize key study findings to date across several broad research areas related to SALURBAL’s aim 1 (for details see Supplement Table 1). Results related to SALURBALs policy evaluation aim (aim2)[12–18] and systems aim (aim 3)[19–21] can be found elsewhere. A full list of SALURBAL Publications is at https://data.lacurbanhealth.org/products/catalog.

### Associations of Urban Environments with Life Expectancy and Cause of Death

#### Life expectancy and causes of death vary significantly across cities in the region, even among cities in the same country

SALURBAL characterized life expectancy (LE) for all SALURBAL cities [6, 22]. We found significant heterogeneity in LE at birth across 363 cities in 9 countries [range 8 years for women (74.4–82.7) and 14 years for men (63.5–77.4)] [6]. This heterogeneity was present not only across countries but also within countries: differences in LE across cities within countries were sometimes as large as 7–10 years. The proportions of deaths from specific causes also varied across cities: some causes of death (unintentional and violent injuries) showed large variation across cities within countries, whereas other causes of death (communicable, maternal, neonatal and nutritional, cancer, cardiovascular disease and other noncommunicable diseases) varied substantially across cities between countries [6].

#### Life Expectancy Also Varies Substantially Within Cities

The exploration of within city differences in health in LMIC remains rare. SALURBAL documented important differences in LE across administrative areas within cities (municipios, comunas or departamentos,) in 6 large cities of the region. Spatial differences in life expectancy at birth within cities (defined as differences between the 90th and the 10th percentile of LE) ranged from 9.8 and 11.2 years in men and women, respectively in Panama City, Panama to 3.9 and 3.0 years in San José, Costa Rica [22]. Analyses investigating even smaller areas (akin to neighborhoods) using spatial Bayesian approaches to accommodate sparse data also found important heterogeneity even within the subcity units [23] [24].

#### City Size has no Unique Relation to Mortality

In contrast to results for 376 cities in the United States in which all-cause mortality is lower in larger than in smaller cities, we found no clear relation between city population size and all-cause mortality in 366 SALURBAL cities [25]. However, associations differed by causes of death. Deaths from cancer, other noncommunicable diseases, and communicable, maternal, neonatal, and nutritional conditions were unrelated to city size in Latin American cities. In contrast, rates of nonviolent injuries, including road traffic deaths and suicides, were lower in larger cities while homicide rates and mortality rates due to sexually transmitted infections, HIV/AIDs, and tuberculosis were generally (although not always) higher in larger cities.

#### Social and Economic Conditions are Associated with Life Expectancy and Cause-Specific Mortality at Multiple Geographic Levels

Differences in city social conditions were linked to differences in life expectancy across cities: a one standard deviation higher social environment index (SEI, reflecting higher population educational attainment, water and sewage access and less overcrowding) was associated with a 0.78 and 0.48-year longer life expectancy in men and women, respectively [6]. In addition, a higher SEI was linked to a lower proportion of deaths from communicable, maternal, neonatal and nutritional deaths but a higher proportion of deaths from cancer, cardiovascular disease and other noncommunicable diseases [6]. Better city socioeconomic conditions were also associated with lower amenable mortality[26], lower infant mortality [27], lower road traffic mortality[28], lower homicides among youth [29], and lower COVID mortality [30].

Socioeconomic conditions are also associated with life expectancy differences across subcity areas (eg municipios) within cities, although the strength of the association differed by city (difference in LE comparing 90th to 10th percentile of area education ranged from 8–11.8 years in Santiago and Panama City to 0.6–0.7 years in San Jose) [22]. Analyses of even smaller areas (neighborhoods) also showed associations of higher area SES with longer life expectancy [8, 24] and with less excess mortality from COVID [31, 32].

#### Cause-Specific Death Rates are Linked to Policy-Relevant Features of Urban Built and Social Environments

Based on hypothesized causal mechanisms and using detailed data on city-level natural, physical and social environments, SALURBAL researchers explored the association of city and neighborhood environmental features with specific causes of death. Road-traffic mortality was higher in cities where urban development was more isolated but lower in cities with higher population density, higher gross domestic product per capita, higher intersection density, and in cities with mass transit [28]. Higher homicide rates among youth and young adults were associated with lower subcity education, lower city GDP, greater isolation of urban development and higher city Gini coefficient [29]. Higher population growth, better living conditions, better service provision, presence of mass transit, and greater women’s empowerment were associated with lower infant mortality [27] [33] [34].

SALURBAL has begun to leverage the longitudinal data compiled by the study. Total annual mortality as well as several cause-specific rates (cardiovascular, cancer, diabetes, respiratory infections, and road traffic injuries) were found to be procyclical (they increase during periods of economic expansions), whereas homicides are countercyclical (they decrease during periods of economic growth) [35]. Using monthly mortality data, SALURBAL researchers found that increases in monthly PM_2.5_ levels are associated with increases in cardiovascular and respiratory mortality [36].

### Associations of Urban Environments with Health and Health Risk Factors

#### Individual-Level Inequities in Health Risk Factors are Pervasive But not Invariant and Differ by Gender and Levels Socioeconomic Development

Analyses of harmonized survey data across hundreds of cities found that self-reported diabetes, measured obesity and self-reported hypertension are strongly and inversely associated with education in women [37–39]. However, associations in men were more variable and sometimes opposite in direction to those observed in women. For example, in men associations of individual-level education with diabetes were inverse (albeit weaker than in women) in Argentina, Brazil, Colombia, Chile, and Mexico, yet, they were positive in Peru, Panama, and El Salvador, and generally became more inverse as city socioeconomic development (indexed by water access, sanitation, overcrowding and education) improved [37]. A similar pattern was observed for obesity and hypertension in men [38] [39]. These findings showing heterogeneity in socioeconomic patterning by gender and broader socioeconomic city context are similar to findings previously reported for countries across the world[40].

An important yet understudied dimension of health inequities In Latin America is the presence of health inequities across racialized groups. An analysis of over 35,000 adults in the 27 state capitals of Brazil found substantial racial disparities in self-rated health and larger disparities in more segregated cities than in less segregated cities for both income and race segregation [41]. In partnership with the Ubuntu Center on Racism, Global Movements and Population Health Equity, SALURBAL conducted an assessment of available data on race and ethnicity in the region and made recommendations for improvement [42].

#### Urban Environments are Related to Measures of Self-Reported Health and Mental Health

Better social conditions and services at the subcity level (a services score indicating access to water and sanitation) and at the city-level (a summary score of services, crowding and education as well as higher GDP) were associated with lower odds of poor self-reported health. [43] [44] Mental health was also found to be associated with city features: in 84 Mexican cities, the presence of more greenspace was associated with lower odds of depressive symptoms [45]. In analyses of 11 cities, longer commuting, experience of traffic delays, commuting by personal vehicle, and worse access to transit were associated with more depressive symptoms [46].

#### Urban Built Environment Features are Related Non-Communicable Disease Risk Factors but Associations are Heterogeneous

Work conducted primarily in high income countries has hypothesized that urban features that signal greater “walkability” can promote walking in daily life and consequently impact physical activity related conditions such as obesity, diabetes, and hypertension. Little research has explored these associations in cities of LMIC. We investigated these associations using over 90,000 survey responses from over 200 cities in 8–10 countries. As hypothesized, living in subcity areas with higher population density was associated with lower rates of diabetes[47] and lower rates of hypertension [48]. Living in greener areas was associated with lower prevalence of BMI, obesity and diabetes [47] and with lower blood pressure [48]. However, contrary to expectation, living in subcity areas with higher intersection density was associated with higher body mass index, obesity, diabetes and hypertension [47, 48]. Findings for city fragmentation were mixed: living in cities where development is more fragmented was associated with higher rates of hypertension [48] but with lower BMI and obesity [47]. It is possible that some of these metrics (eg intersection density and fragmentation) are confounded by other factors or simply are not as important as predictors of physical activity in cities were a significant amount of the population already walks by necessity as part of their commute [49]. These results highlight the importance of examining these relationships in various settings.

Taking advantage of rich local data, SALURBAL examined impacts of the food environment on health risk factors in Mexico. Larger increases in the density of fruit and vegetable stores over time were associated with declines or slower increases in BMI over time among children [50]. We also found higher odds of diabetes and higher blood pressure in adults living in municipalities where fruits and vegetable stores had decreased, or where convenience stores or supermarkets had increased over time [51] [52].

Many Latin American cities are characterized by high levels of traffic congestion. We found that travel time and travel delay were both associated with lower odds of frequent vegetable consumption. In addition, longer travel delay time was associated with more frequent consumption of sugar sweetened beverages [53]. However we found no association between city-level travel time an odds of obesity or diabetes, perhaps because it is difficult to isolate the impact of travel from the many other lifecourse factors influencing risks of obesity and diabetes in these contexts [54].

### Urban Policies are Associated with Heterogeneous Urban Environments

SALURBAL used finite mixture modeling to identify city profiles based on landscape and street design metrics. We identified four profiles for landscape and four for the street design domain [55]. We investigated whether certain city landscape profiles were more likely to show positive or negative health and environmental co-benefits. Cities with the scattered pixels profile (representing low fragmentation, high isolation, and more compact development) were more likely than other city profiles to have positive co-benefits. In contrast, the contiguous large inkblot cities (higher fragmentation and complex shape, and often very large) were the least likely to be in the positive co-benefits class [56]. These results highlight the complex ways in which urban form can be linked to health and environmental co-benefits.

SALURBAL also developed an extensive characterization of green space and parks [57]. [58] Most SALURBAL cities experienced increases in greenness over time but cities with higher SES experienced larger increases in greenness [59]. There was some evidence that less greenness was associated with faster warming over time and that recent greening may lessen warming [60].

SALURBAL analyses described transportation-related features of Latin American cities. In an analysis of 300 cities we found a that the average car rate (number of passenger vehicles per 1000 inhabitants) increased by 30% between 2010 and 2015 (from 210.2 cars per 1000 inhabitants in 2010 to 273.3 care per 1000 inhabitants in 2015). Development fragmentation, urban form complexity and circuity of the street network were associated with higher motorization rates, whereas higher population density was associated with less pronounced increases in motorization over time [61]. We also found that despite increases in motorization, walking remains a major mode of transportation in Latin American cities [62], often driven by necessity, highlighting the need to make public transportation both accessible and safe to reduce excessive walking times while still promoting physical activity. We also examined population levels and trends in cycling. Persons with higher SES had lower odds of using bicycles but odds of bicycle use have increased over time, especially among high SES populations [63] emphasizing the need for policies to increase active travel to focus across social groups.

SALURBAL also characterized levels and drivers of air pollution in cities of the region. In analyses of PM2.5 levels across 366 cities[64] we found that about 172 million or 58% of the population lived in areas with air pollution levels above the then defined WHO-AQG of 10 μg/m^3^ annual average. Larger cities, cities with higher GDP, higher motorization rate and higher congestion tended to have higher PM_2.5_. Cities with higher population density and with mass transit had lower levels of PM_2.5_. At the sub-city level, higher intersection density was associated with higher PM_2.5_ and more green space was associated with lower PM_2.5_ [64]. We also found that 85% of residents lived in neighborhoods with ambient annual NO_2_ above WHO guidelines [65]. At the city level, higher vehicle congestion, population size, and population density were associated with higher ambient NO_2_ whereas higher neighborhood-level greenness were associated with lower ambient NO_2_. These results for two important pollutants highlight the potential role of urban policies in reducing exposures.

### Climate Change as an Emerging Health Threat in Urban Areas of the Region

In an analysis of 326 cities we found that a substantial proportion of deaths is attributable to non-optimal ambient temperatures and that marginal increases in observed hot temperatures are associated with steep increases in mortality risk with stronger associations observed in older persons [7]. We also found that higher temperatures during gestation (especially during months 7–9) were associated with lower birthweight. [66] Greater greenness was found to provide modest protection against heat-related mortality particularly in arid climate zones [67].

## Discussion

SALURBAL Aim 1 analyses have yielded several significant findings. First, Latin American cities and neighborhoods within them reveal significant heterogeneity in health. Second, socioeconomic inequities across cities as well as across neighborhoods within cities are important predictors of differences in health across and within cities. Third, individual-level inequities in health are substantial but differ by gender and inverse associations of individual level SES with health emerge or become stronger as city or country socioeconomic development increases. Fourth, policy relevant city features including features of the built and transportation environment amenable to urban planning policies and social factors amenable to social and economic policy impact specific health outcomes, and also impact environmental exposures (such as air pollution levels) that have been shown to have significant health effects. These sets of findings highlight significant ways in which urban policies can impact health and health equity in cities of LMICs.

The SALURBAL experience has shown that a large collaborative, multicountry partnership can be simultaneously participatory, scientifically rigorous and productive, and promote meaningful local engagement and regional capacity building. The SALURBAL project has published 137 papers in peer-reviewed journals to date, 58% of them led by investigators from the region and 88% led by junior/new investigators. SALURBAL facilitated the creation of a team and governance structure that meaningfully engaged local teams, understood local challenges, and leveraged the infrastructure and resources available in high income countries (in this case in the US) in direct support of the regional collaboration. The breadth of SALURBAL allowed the study to be goal and product oriented and yet flexible and opportunistic.

SALURBAL has been extraordinarily impactful in at least two ways. First, the study has generated a comprehensive set of findings describing levels and variations in urban health across the region with a focus on inequities. It illustrated how country and city level factors interact with individual-level factors (such as education) in shaping inequities, highlighting the impact of city social and economic policies. It has also documented how a range of policy amenable factors (including features of social, natural and built environments) relate to health and could therefore be leveraged via multisectoral policies to improve health. Second, through its data compilation/harmonization and through its collaborative approach to paper writing it has built capacity in data and analysis, scientific writing and dissemination, and meaningful multi-country collaboration across the region, in ways that can translate to other scientific questions.

Two key strategies contributed to SALURBAL’s success in achieving its first aim. The first key strategy was the creation of a data resource that was comprehensive in its geographic and temporal span and in the domains encompassed and yet flexible, allowing investigators to slot in different types of data according to availability. In compiling this data resource SALURBAL used a number of strategies and integrated a variety of sources in an opportunistic manner including (1) data that was available in a standardized manner across all countries and that could be processed in a systematic way to obtain measures for areas of various size (eg temperature and air pollution data, see Table 2); (2) data that was routinely collected by government agencies but that could be harmonized relatively easily using standardized approaches such as those adopted by prior international collaborations such as the Integrated Public Use MicroData Series (IPUMS) or other published global analyses of mortality data (census data and vital statistics) [6, 28]; and (3) data from surveys conducted by national agencies for surveillance or other purposes that were often quite different in survey questions and in sampling approaches but for which some harmonization (or harmonization across some countries) was possible [9]. A key lesson was that with time and resources, including strong engagement from countries in the leadership and implementation of the project, it is feasible to compile and process existing data from LMIC in ways that allow comparative analyses.

A second strategy fundamental to the success of SALURBAL Aim 1 was the creation of an organizational structure and a set of systems and processes that facilitated collaboration and scientific rigor, including formal and informal supports for applied capacity building. This required substantial investment of time and effort in developing, communicating and implementing clear processes and guidelines for collaboration, regular meetings of multiple groups engaging partners across the region, a robust leadership team to oversee, guide and troubleshoot, and communication and outreach strategies to facilitate engagement of a very diverse group across multiple countries with varying levels of expertise and experience. The SALURBAL experience shows that meaningful multicountry research that fully engages and builds capacity in institutions from LMIC is feasible if appropriate governance and collaborative and support systems are put in place.

The study also faced a number of challenges. A first challenge was the definition of “cities”. Recognizing that there is no unique way to define cities, SALURBAL adopted a practical approach initially driven by data availability, but also created alternative more precise definitions based on built up areas [4]. However not all of the city definitions could be linked to available health data and the ones that could often include some areas that were not built up. In addition some of the definitions were not directly aligned with political responsibility or spanned more than one political jurisdiction (eg a city core and surrounding urbanized areas). Thus in operationalizing “cities” SALURBAL needed to balance theoretical relevance (eg using the broader urban agglomeration because of the interconnected nature of its various components), practicalities related to data linkage (some theoretically relevant definitions could not be linked to health data), and political relevance (direct implications for policy).

A second challenge was heterogeneity in data availability and quality. SALURBAL took a flexible approach to data compilation, incorporating as much data as possible (as long as it met basic quality requirements) but also noting quality issues and caveats in documentation. A range of strategies were deployed to address data challenges that emerged (Table 3). The team built many partnerships with local governmental data agencies to access official data, expand capacity to use the data and improve the data. SALURBAL also issued briefs on data availability and quality [68], improved data using state of the art methods [6] and also highlighted important data gaps [42]. The study faced challenges related to data access and the absence of a tradition of public data sharing. In many cases this was overcome thanks to the initiative of local SALURBAL team members, the development of flexible data use agreements that were used to accommodate agency requirements whenever possible, and the development of partnerships with local agencies including the return of processed data and the invitation to engage in workshops or other activities hosted by SALURBAL. In many cases geographic identifiers smaller than SALURBAL subcities were not available. SALURBAL was able to directly support additional georeferencing thus providing a service to agencies that would subsequently share data with SALURBAL. A complex set of Data Use Agreements were required to implement a range of agreements that varied by data sources and by country, and even over time. By using what is available and encouraging cross-country comparisons, SALURBAL aimed to make visible what can actually be done with existing data and highlight why having additional accessible high quality data is important.

A third challenge was variability in the research experience of the team. SALURBAL purposefully engaged a wide range of researchers and trainees across the region and both formal and informal capacity building was embedded into all activities from leadership, to data processing, to paper writing to dissemination and outreach. This sometimes meant that publications required more time for completion. The study promoted a decentralized and bottom-up approach to identification of research questions while also ensuring that key questions were addressed via guidance on priority papers by the study executive committee. As a result, SALURBAL produced a range of scientific publications some more directly aligned with its original goals and others more tangential to those goals but also important as they reflected the priorities and interests of the broader research team. Recognizing the importance of disseminating the work SALURBAL has also published in a wide range of journals, including regional journals.

A fourth challenge was related to the generation of timely evidence relevant to local action. Compiling, harmonizing and analyzing data by a collaborative team takes time. SALURBAL addressed this challenge by generating descriptive information as quickly as possible and by also initially focusing on reviewing and disseminating what was already known. [69] [70] A related challenge was balancing results generalizable to Latin American cities as a whole (and derived from an analysis of all cities, a big strength of SALURBAL) with locally-specific analyses that may be more motivating to local policy makers. SALURBAL embraced both the generation of evidence that is relevant across cities (e.g. [6, 28].) as well as country and even city specific analyses (e..g [24, 52]).

A project of this breadth and level of regional engagement requires significant resources. Wellcome Trust funding allowed us to establish a critical coordination infrastructure and data resource, support investigators and staff across the region, engage in capacity-strengthening, and invest in dissemination and outreach efforts. The funding also allowed us to be flexible, strategic and opportunistic. Institutions involved in the project provided cost sharing in various ways. We were also able to obtain ancillary funding from other sources (regional, US and global) to support extensions of the project. Most importantly, the culture of collaboration firmly grounded in the region, with multiple opportunities for exchange (in person and remotely) and a commitment to overcoming obstacles and “making the impossible possible” was fundamental to the project’s success.

SALURBAL has made remarkable progress but there is more to do. There are significant opportunities to expand use of the data resource by conducting longitudinal analyses, spatially explicit within city analyses, and policy evaluation. The growing aging populations of LMICs will need to receive increasing attention in urban health studies. The collaborative platform created by SALURBAL can be leveraged to support additional capacity building and increase the diversity of the team by deliberately engaging and supporting investigators from groups underrepresented in the research teams. SALURBAL is also working to enhance the utility of data and findings for policy making by developing new dissemination tools such as city profiles and interactive and dynamic ways to share findings. New funding will also allow a climate focused SALURBBAL (SALURBAL-Climate) to focus on quantifying climate change health impacts and identifying effective mitigation and adaptation policies in cities of the region.

Lastly it is critical to continue to build capacity across institutions of the region not only in conducting and disseminating research but also in coordinating and leading large regional partnerships of this type. SALURBAL has shown the immense value of south-south collaborations in strengthening local capacity, while also highlighting how the resources and expertise in high income countries can be leveraged to support the goal of meaningful regional engagement that is not extractive. Ensuring long term sustainability of the platform and collaborative approach with increasing leadership by institutions in the region is the next major challenge for SALURBAL.

## Supplementary Information

Below is the link to the electronic supplementary material.

Supplementary file1 (DOCX 113 KB)

## Data Availability

Data is available at https://data.lacurbanhealth.org/.

## References

[1] United Nations, Department of Economic and Social Affairs, Population Division. *The World’s Cities in 2018—Data Booklet (ST/ESA/ SER.A/417)*. 2018.

[2] Diez Roux AV, Slesinski SC, Alazraqui M, et al. A Novel International Partnership for Actionable Evidence on Urban Health in Latin America: lAC-Urban Health and SALURBAL. *Global Chall*. 2019;3(4):1800013–1800013. 10.1002/gch2.201800013 PMC6450446 31565372

[3] Baquero S, Montes F, Stankov I, et al. Assessing cohesion and diversity in the collaboration network of the SALURBAL project. *Sci Rep*. 2023;13(1):1–13. 37165002 10.1038/s41598-023-33641-x PMC10172186

[4] Quistberg DA, Diez Roux AV, Bilal U, et al. Building a Data Platform for Cross-Country Urban Health Studies: the SALURBAL Study. *J Urban Health*. 2019;96(2):311. 30465261 10.1007/s11524-018-00326-0 PMC6458229

[5] SALURBAL. SALURBAL Portal. (n.d.). Available at: https://data.lacurbanhealth.org/. Accessed 1 Nov 2024.

[6] Bilal U, Hessel P, Perez-Ferrer C, et al. Life expectancy and mortality profiles are highly heterogeneous in 363 cities of Latin America: the SALURBAL project. *Nat Med*. 2021;2021(27):463–70. 10.1038/s41591-020-01214-4 PMC7960508 33495602

[7] Kephart JL, Sánchez BN, Moore J, et al. City-level impact of extreme temperatures and mortality in Latin America. *Nat Med*. 2022;28(8):1700–5. 35760859 10.1038/s41591-022-01872-6 PMC9388372

[8] Rodríguez López S, Tumas N, Bilal U, et al. Intraurban socioeconomic inequalities in life expectancy: a population-based cross-sectional analysis in the city of Córdoba, Argentina (2015–2018). *BMJ Open*. 2022;12(9):e061277. 36691155 10.1136/bmjopen-2022-061277 PMC9442478

[9] Moore K, Lazo M, Ortigiza A, et al. *Data resource profile: Harmonized health survey data for 240 cities across 11 countries in Latin America: the SALURBAL Project*. *IJE*. (in press). 10.1093/ije/dyae171 PMC11703366 39761604

[10] Wilkinson MD, Dumontier M, Aalbersberg IJ, et al. The FAIR Guiding Principles for scientific data management and stewardship. *Sci Data*. 2016;3(1):160018. 26978244 10.1038/sdata.2016.18 PMC4792175

[11] Slesinski SC, Indvik K, Barrientos-Gutierrez T, et al. Research Translation to Promote Urban Health in Latin America: The SALURBAL Experience. *J Urban Health*. 2024;2024:1–18. 10.1007/s11524-024-00877-5 PMC11652544 38935205

[12] Morales-Betancourt R, Wilches-Mogollon MA, Sarmiento OL, et al. Commuter’s personal exposure to air pollutants after the implementation of a cable car for public transport: Results of the natural experiment TrUST. *Sci Total Environ*. 2023;865:160880–160880. 36516922 10.1016/j.scitotenv.2022.160880 PMC7616957

[13] Baldovino-Chiquillo L, Sarmiento OL, O’Donovan G, et al. Effects of an urban cable car intervention on physical activity: the TrUST natural experiment in Bogotá. *Colomb Lancet Glob Health*. 2023;11(8):e1290–300. 10.1016/S2214-109X(23)00274-7 PMC10369015 37474235

[14] Baeza F, Vives Vergara A, González F, et al. The Regeneración Urbana, Calidad de Vida y Salud - RUCAS project: a Chilean multi-methods study to evaluate the impact of urban regeneration on resident health and wellbeing. *BMC Public Health*. 2021;21(1):1–15. 33858373 10.1186/s12889-021-10739-3 PMC8047526

[15] Quintero Valverde C, Perez-Ferrer C, ChiasBecerril L, et al. Evaluation of road safety policies and their enforcement in Mexico City, 2015–2019: an interrupted time-series study. *Inj Preve J Int Soc Child Adolesc Inj Prev*. 2022;29(1):35–41. 10.1136/ip-2022-044590 PMC7614109 36096653

[16] Saavedra-Garcia L, Moscoso-Porras M, Diez-Canseco F. An Experimental Study Evaluating the Influence of Front-of-Package Warning Labels on Adolescent’s Purchase Intention of Processed Food Products. *Int J Environ Res Publ Health*. 2022;19(3):1094. 10.3390/ijerph19031094 PMC8833989 35162126

[17] Mullachery PH, Quistberg DA, Lazo M, et al. Evaluation of the national sobriety checkpoints program in Mexico: a difference-in-difference approach with variation in timing of program adoption. *Inj Epidemi*. 2022;9(1):32. 10.1186/s40621-022-00407-4 PMC9680121 36411475

[18] Guzman LA, Arellana J, Sarmiento OL. Time use decisions in vulnerable urban communities when implementing innovative transport alternatives. *Travel Behav Soc*. 2024;36:100806.

[19] Langellier BA, Kuhlberg JA, Ballard EA, et al. Using community-based system dynamics modeling to understand the complex systems that influence health in cities: The SALURBAL study. *Health Place*. 2019;60:102215–102215. 31586769 10.1016/j.healthplace.2019.102215 PMC6919340

[20] Langellier BA, Stankov I, Hammond RA, et al. Potential impacts of policies to reduce purchasing of ultra-processed foods in Mexico at different stages of the social transition: an agent-based modelling approach. *Public Health Nutr*. 2022;25(6):1711–9. 34895382 10.1017/S1368980021004833 PMC7612742

[21] Stankov I, Meisel JD, Sarmiento OL, et al. Uncovering physical activity trade-offs in transportation policy: A spatial agent-based model of Bogota, Colombia. *Int J Behav Nutr Phys Act*. 2024;21(1):54. 38720323 10.1186/s12966-024-01570-1 PMC11077730

[22] Bilal U, Alazraqui M, Caiaffa WT, et al. Inequalities in life expectancy in six large Latin American cities from the SALURBAL study: an ecological analysis. *Lancet Planet Health*. 2019;3(12):e503–10. 31836433 10.1016/S2542-5196(19)30235-9 PMC6926471

[23] Rodríguez López S, Bilal U, Ortigoza AF, Diez-Roux AV. Educational inequalities, urbanicity and levels of non-communicable diseases risk factors: evaluating trends in Argentina (2005–2013). *BMC Public Health*. 2021;21(1):1–12. 34416876 10.1186/s12889-021-11617-8 PMC8379776

[24] Trotta A, Bilal U, Acharya B, et al. Spatial Inequities in Life Expectancy in Small Areas of Buenos Aires Argentina 2015–2017. *J Urban Health*. 2023;100(3):577–90. 37225944 10.1007/s11524-023-00730-1 PMC10323071

[25] Bilal U, de Castro CP, Alfaro T, et al. Scaling of mortality in 742 metropolitan areas of the Americas. *Sci Adv*. 2021;7(50):1–13. 10.1126/sciadv.abl6325 PMC8654292 34878846

[26] Mullachery PH, Rodriguez DA, Miranda JJ, et al. Mortality amenable to healthcare in Latin American cities: a cross-sectional study examining between-country variation in amenable mortality and the role of urban metrics. *Int J Epidemiol*. 2022;51(1):303–13. 34339492 10.1093/ije/dyab137 PMC8856009

[27] Ortigoza AF, Tapia Granados JA, Miranda JJ, et al. Characterising variability and predictors of infant mortality in urban settings: findings from 286 Latin American cities. *J Epidemiol Community Health*. 2021;75(3):264–70. 33060193 10.1136/jech-2020-215137 PMC7892385

[28] Quistberg DA, Hessel P, Rodriguez DA, et al. Urban landscape and street-design factors associated with road-traffic mortality in Latin America between 2010 and 2016 (SALURBAL): an ecological study. *Lancet Planet Health*. 2022;6(2):e122–31. 35150622 10.1016/S2542-5196(21)00323-5 PMC8850369

[29] de Lima Friche AA, Silva UM, Bilal U, et al. Variation in youth and young adult homicide rates and their association with city characteristics in Latin America: the SALURBAL study. *Lancet Reg health Am*. 2023;20:100476–100476. 36970493 10.1016/j.lana.2023.100476 PMC10033737

[30] de Sousa Filho JF, Silva UM, Lima LL, et al. Association of urban inequality and income segregation with COVID-19 mortality in Brazil. *PLoS ONE*. 2022;17(11):e0277441. 36378655 10.1371/journal.pone.0277441 PMC9665357

[31] Alfaro T, Martinez-Folgar K, Vives A, Bilal U. Excess Mortality during the COVID-19 Pandemic in Cities of Chile: Magnitude Inequalities and Urban Determinants. *J Urban Health*. 2022;99:0123456789. 10.1007/s11524-022-00658-y PMC9187147 35688966

[32] Perner MS, Trotta A, Bilal U, et al. Social inequalities and COVID-19 mortality between neighborhoods of Bariloche city, Argentina. *Int J Equity Health*. 2023;22(1):1–11. 37770868 10.1186/s12939-023-02019-w PMC10537962

[33] Ortigoza A, Braverman A, Hessel P, et al. Women’s empowerment and infant mortality in Latin America: evidence from 286 cities. *Cities Health*. 2021;00(00):1–9. 10.1080/23748834.2021.1908794 PMC7614198 36818398

[34] Hessel P, Jaramillo MJG, Rasella D, Duran AC, Sarmiento OL. Increases in women’s political representation associated with reductions in child mortality in Brazil. *Health Aff*. 2020;39(7):1166–1166. 10.1377/hlthaff.2019.01125 PMC7610598 32634348

[35] Leveau CM, Tapia Granados JA, Dos Santos MI, Castillo-Riquelme M, Alazraqui M. Are Wealthier Times Healthier in Cities? Economic Fluctuations and Mortality in Urban Areas of Latin America. *Int J Public Health*. 2021;66:1–9. 10.3389/ijph.2021.1604318 PMC8696345 34955702

[36] Gouveia N, Rodriguez-Hernandez JL, Kephart JL, et al. Short-term associations between fine particulate air pollution and cardiovascular and respiratory mortality in 337 cities in Latin America. *Sci Total Environ*. 2024;10(920):171073. 10.1016/j.scitotenv.2024.171073 PMC10918459 38382618

[37] Braverman-Bronstein A, Hessel P, González-Uribe C, et al. Association of education level with diabetes prevalence in Latin American cities and its modification by city social environment. *J Epidemiol Community Health*. 2021;75(9):874–80. 33542029 10.1136/jech-2020-216116 PMC7611487

[38] Mazariegos M, Auchincloss AH, Braverman-Bronstein A, et al. Educational inequalities in obesity: a multilevel analysis of survey data from cities in Latin America. *Public Health Nutr*. 2022;25(7):1790–8. 34167613 10.1017/S1368980021002457 PMC7613035

[39] Coelho DM, de Souza Andrade AC, Silva UM, et al. Gender differences in the association of individual and contextual socioeconomic status with hypertension in 230 Latin American cities from the SALURBAL study: a multilevel analysis. *BMC Public Health*. 2023;23(1):1–11. 37568082 10.1186/s12889-023-16480-3 PMC10416382

[40] Cohen AK, Rai M, Rehkopf DH, Abrams B. Educational attainment and obesity: a systematic review. *Obes Rev*. 2013;14(12):989–1005. 23889851 10.1111/obr.12062 PMC3902051

[41] Guimarães JMN, Yamada G, Barber S, et al. Racial Inequities in Self-Rated Health Across Brazilian Cities: does Residential Segregation Play a Role? *Am J Epidemiol*. 2022;191(6):1071–80. 35244147 10.1093/aje/kwac001 PMC9169054

[42] Bashir H, Ferreira A, Ortigoza A, Carabili M, Ramos D, Slesinski C, Goes E, Barber S. *Making the invisible visible: Race racism and health data lessons from Latin American Countries. *2023. Available at https://drexel.edu/lac/data-evidence/briefs/.

[43] Teixeira Vaz C, Moraes Coelho D, Moreira Silva U, et al. Social environment characteristics are related to self-rated health in four Latin America countries: evidence from the SALURBAL Project. *Health Place*. 2023;83:1353–8292. 10.1016/j.healthplace.2023.103110 PMC10561099 37708687

[44] Castillo-Riquelme M, Yamada G, Diez Roux AV, et al. Aging and self-reported health in 114 Latin American cities: gender and socio-economic inequalities. *BMC Public Health*. 2022;22(1):1–14. 35932016 10.1186/s12889-022-13752-2 PMC9356475

[45] Bakhtsiyarava M, Ju Y, Moran M, et al. Associations between urban greenspace and depressive symptoms in Mexico’s cities using different greenspace metrics. *Appl Geogr*. 2024;164:103219.

[46] Wang X, Rodríguez DA, Sarmiento OL, Guaje O. Commute patterns and depression: evidence from eleven Latin American cities. *J Transp Health*. 2019;14:100607–100607. 31853443 10.1016/j.jth.2019.100607 PMC6894323

[47] Anza-Ramirez C, Lazo M, Zafra-Tanaka JH, et al. The urban built environment and adult BMI, obesity, and diabetes in Latin American cities. *Nat Commun*. 2022;13(1):1–9. 36581636 10.1038/s41467-022-35648-w PMC9800402

[48] Avila-Palencia I, Rodríguez DA, Miranda JJ, et al. Associations of Urban Environment Features with Hypertension and Blood Pressure across 230 Latin American Cities. *Environ Health Perspect*. 2022;130(2):1–10. 10.1289/EHP7870 PMC8846315 35167325

[49] Salvo D, Jauregui A, Adlakha D, Sarmiento OL, Reis RS. When Moving Is the Only Option: the Role of Necessity Versus Choice for Understanding and Promoting Physical Activity in Low- and Middle-Income Countries. *Annu Rev Public Health*. 2023;44:151–69. 36525957 10.1146/annurev-publhealth-071321-042211

[50] Ramírez-Toscano Y, Pérez-Ferrer C, Bilal U, Auchincloss AH, Barrientos-Gutierrez T. Longitudinal association between density of retail food stores and body mass index in Mexican school children and adolescents. *Int J Obesity*. 2003;47(5):365–74. 10.1038/s41366-023-01273-w PMC10147568 36792910

[51] Pérez-Ferrer C, Auchincloss AH, Barrientos-Gutierrez T, et al. Longitudinal changes in the retail food environment in Mexico and their association with diabetes. *Health Place*. 2020;66:102461. 33039800 10.1016/j.healthplace.2020.102461 PMC7705211

[52] Armendariz M, Pérez-Ferrer C, Basto-Abreu A, Lovasi GS, Bilal U, Barrientos-Gutiérrez T. Changes in the Retail Food Environment in Mexican Cities and Their Association with Blood Pressure Outcomes. *Int J Environ Res Publ Health*. 2022;19(3):1353. 10.3390/ijerph19031353 PMC8834862 35162376

[53] Guimarães JMN, Acharya B, Moore K, et al. City-Level Travel Time and Individual Dietary Consumption in Latin American Cities: Results from the SALURBAL Study. *Int J Environ Res Publ Health*. 2022;19(20):13443. 10.3390/ijerph192013443 PMC9602577 36294020

[54] Delclòs-Alió X, Rodríguez DA, Olmedo NL, et al. Is city-level travel time by car associated with individual obesity or diabetes in Latin American cities? Evidence from 178 cities in the SALURBAL project. *Cities*. 2022;131:103899. 36277810 10.1016/j.cities.2022.103899 PMC7613723

[55] Sarmiento OL, Useche AF, Rodriguez DA, et al. Built environment profiles for Latin American urban settings: The SALURBAL study. *PLoS ONE*. 2021;16(10):e0257528. 34699532 10.1371/journal.pone.0257528 PMC8547632

[56] Avila-Palencia I, Sánchez BN, Rodríguez DA, et al. Health and Environmental Co-Benefits of City Urban Form in Latin America: An Ecological Study. *Sustainability*. 2022;14(22):14715–14715. 36926000 10.3390/su142214715 PMC7614319

[57] Ju Y, Dronova I, Delclòs-Alió X. A 10 m resolution urban green space map for major Latin American cities from Sentinel-2 remote sensing images and OpenStreetMap. *Sci Data*. 2022;9(1):1–9. 36153342 10.1038/s41597-022-01701-y PMC9509366

[58] Slovic AD, Kanai C, Marques Sales D, et al. Spatial data collection and qualification methods for urban parks in Brazilian capitals: an innovative roadmap. *PLoS ONE*. 2023;18(8):e0288515. 37561781 10.1371/journal.pone.0288515 PMC10414613

[59] Ju Y, Moran M, Wang X, et al. Latin American cities with higher socioeconomic status are greening from a lower baseline: Evidence from the SALURBAL project. *Environ Res Lett*. 2021;16(10):104052. 34691242 10.1088/1748-9326/ac2a63 PMC8524204

[60] Ju Y, Dronova I, Rodriguez DA, Bakhtsiyarava M, Farah I. Recent greening may curb urban warming in Latin American cities of better economic conditions. *Landsc Urban Plan*. 2023;240:104896–104896. 10.1016/j.landurbplan.2023.104896 PMC10570748 38046954

[61] Delclòs-Alió X, Kanai C, Soriano L, et al. Cars in Latin America: An exploration of the urban landscape and street network correlates of motorization in 300 cities. *Travel Behav Soc*. 2023;30:192–201.

[62] Delclòs-Alió X, Rodríguez DA, Medina C, et al. Walking for transportation in large Latin American cities: walking-only trips and total walking events and their sociodemographic correlates. *Transp Rev*. 2022;42(3):296–317. 35431369 10.1080/01441647.2021.1966552 PMC7612619

[63] Avila-Palencia I, Sarmiento OL, Gouveia N, et al. Bicycle use in Latin American cities: changes over time by socio-economic position. *Front Sustain Cities*. 2023;5:1055351–1055351. 39639923 10.3389/frsc.2023.1055351 PMC7616888

[64] Gouveia N, Kephart JL, Dronova I, et al. Ambient fine particulate matter in Latin American cities: levels, population exposure, and associated urban factors. *Sci Total Environ*. 2021;772:145035–145035. 33581538 10.1016/j.scitotenv.2021.145035 PMC8024944

[65] Kephart JL, Gouveia N, Rodriguez DA, et al. Ambient nitrogen dioxide in 47 187 neighbourhoods across 326 cities in eight Latin American countries: population exposures and associations with urban features. *Lancet Planet Health*. 2023;7(12):e976–84. 38056968 10.1016/S2542-5196(23)00237-1 PMC10716820

[66] Bakhtsiyarava M, Ortigoza A, Sánchez BN, et al. Ambient temperature and term birthweight in Latin American cities. *Environ Int*. 2022;167:107412. 35870377 10.1016/j.envint.2022.107412 PMC9376808

[67] Schinasi LH, Bakhtsiyarava M, Sanchez BN, et al. Greenness and excess deaths from heat in 323 Latin American cities: Do associations vary according to climate zone or green space configuration? *Environ Int*. 2023;180:108230–108230. 37776620 10.1016/j.envint.2023.108230 PMC10594062

[68] SALURBAL. SALURBAL Data Briefs. Available at: https://drexel.edu/lac/data-evidence/data/. Accessed 1 Nov 2024.

[69] Sarmiento OL, Siri J, Rodriguez D, Higuera-Mendieta D, Gonzalez S, Montero S, Barrientos T, Morales R, Mora R, Slesinski C, Diez Roux AV. *Sustainable transport and urban health: lessons from Latin American cities*. 2017. Available at https://drexel.edu/lac/data-evidence/briefs/.

[70] SALURBAL. SALURBAL Policy Briefs. Available at: https://drexel.edu/lac/data-evidence/briefs/. Accessed 1 Nov 2024.

[71] Santos MId, Santos GFd, Freitas A, et al. Urban income segregation and homicides: An analysis using Brazilian cities selected by the Salurbal project. *SSM Popul Health*. 2021;14:100819. 34041354 10.1016/j.ssmph.2021.100819 PMC8142279

[72] Trejo B, Michael Y, Diez Roux AV, et al. *Characterizing the killing of girls and women in urban settings in Latin America, 2000–2019: an analysis of variability and time trends using mortality data from vital registration systems*. BMJ Public Health. 2024. 10.1136/bmjph-2024-000985 PMC11812913 40018091

[73] Henson RM, Mullachery PH, Sanchez-Pajaro A, et al. Spatial Heterogeneity in Fatal Overdose Rate Trends in Mexican Cities: 2005–2021. *Am J Public Health*. 2024;114(7):705–13. 38723222 10.2105/AJPH.2024.307650 PMC11153949

[74] Moran MR, Bilal U, Dronova I, et al. The equigenic effect of greenness on the association between education with life expectancy and mortality in 28 large Latin American cities. *Health Place*. 2021;72:102703. 34753000 10.1016/j.healthplace.2021.102703 PMC8633763

[75] Carvajal GA, Sarmiento OL, Medaglia AL, et al. Bicycle safety in Bogotá: A seven-year analysis of bicyclists’ collisions and fatalities. *Accid Anal Prev*. 2020;144:105596–105596. 32603927 10.1016/j.aap.2020.105596 PMC7447975

[76] Bilal U, Alfaro T, Vives A. COVID-19 and the worsening of health inequities in Santiago. *Chile Int J Epidemiol*. 2021;50(3):1038–40. 33537771 10.1093/ije/dyab007 PMC7928917

[77] Braverman-Bronstein A, Vidaña-Pérez D, Ortigoza AF, et al. Adolescent birth rates and the urban social environment in 363 Latin American cities. *BMJ Glob Health*. 2022;7(10):9737–9737. 10.1136/bmjgh-2022-009737 PMC9577896 36253017

[78] Braverman-Bronstein A, Ortigoza AF, Vidaña-Pérez D, et al. Gender inequality, women’s empowerment and adolescent birth rates in 363 Latin American cities. *Soc Sci Med*. 2023;317:115566–115566. 36446141 10.1016/j.socscimed.2022.115566 PMC7613905

[79] Braverman-Bronstein A, Vidaña-Pérez D, Diez Roux AV, Pérez Ferrer C, Sánchez BN, Barrientos-Gutiérrez T. Association of service facilities and amenities with adolescent birth rates in Mexican cities. *BMC Public Health*. 2023;23(1):1–10. 37430299 10.1186/s12889-023-16251-0 PMC10334546

[80] Perner MS, Ortigoza A, Trotta A, et al. Cesarean sections and social inequalities in 305 cities of Latin America. *SSM Popul Health*. 2022;19:101239–101239. 36203470 10.1016/j.ssmph.2022.101239 PMC9529579

[81] Rodríguez López S, Tumas N, Ortigoza A, de Lima Friche AA, Diez-Roux AV. Urban social environment and low birth weight in 360 Latin American cities. *BMC Public Health*. 2021;21(1):1–10. 33902522 10.1186/s12889-021-10886-7 PMC8073945

[82] Valentino G, Ortigoza A, Rodriguez Osiac L, Doberti T, Mullachery P, Nazzal C. Smoking Ban Law in Chile: Impact in Newborns’ Birth Weight by Women’s Age Groups and by City Population Density. *Int J Public Health*. 2022;67:1605087. 36579137 10.3389/ijph.2022.1605087 PMC9791390

[83] Huynh TB, Oddo VM, Trejo B, et al. Association between informal employment and depressive symptoms in 11 urban cities in Latin America. *SSM Popul Health*. 2022;18:101101–101101. 35698484 10.1016/j.ssmph.2022.101101 PMC9187523

[84] Alpaugh V, Ortigoza A, Braverman Bronstein A, et al. Association Between Household Deprivation and Living in Informal Settlements and Incidence of Diarrhea in Children Under 5 in Eleven Latin American Cities. *J Urban Health*. 2024;101(3):629–37. 38652338 10.1007/s11524-024-00854-y PMC11189882

[85] Tumas N, Rodríguez López S, Bilal U, Ortigoza AF, Diez Roux AV. Urban social determinants of non-communicable diseases risk factors in Argentina. *Health Place*. 2022;77:102611. 34210611 10.1016/j.healthplace.2021.102611 PMC8714870

[86] Zafra-Tanaka JH, Braverman A, Anza-Ramirez C, et al. City features related to obesity in preschool children: a cross-sectional analysis of 159 cities in six Latin American countries. *Lancet Reg Health Am*. 2023;20:100458. 36942152 10.1016/j.lana.2023.100458 PMC10023940

[87] Tumas N, Rodríguez López S, Mazariegos M, et al. Are Women’s Empowerment and Income Inequality Associated with Excess Weight in Latin American Cities? *J Urban Health Bull N Y Acad Med*. 2022;99(6):1091–1091. 10.1007/s11524-022-00689-5 PMC7613896 36357625

[88] Moran MR, Rodríguez DA, Cotinez-O’Ryan A, Miranda JJ. Park use, perceived park proximity, and neighborhood characteristics: evidence from 11 cities in Latin America. *Cities*. 2019;2020(105):102817. 10.1016/j.cities.2020.102817 PMC7490577 33012941

[89] Cunha MdCM, Ju Y, Morais MHF, et al. Disentangling associations between vegetation greenness and dengue in a Latin American city: Findings and challenges. *Landsc Urban Plan*. 2021;216:104255. 10.1016/j.landurbplan.2021.104255 PMC8519391 34675450

[90] Kephart JL, Delclòs-Alió X, Rodríguez DA, et al. The effect of population mobility on COVID-19 incidence in 314 Latin American cities: a longitudinal ecological study with mobile phone location data. *The Lancet Digital Health*. 2021;3(11):e716–22. 34456179 10.1016/S2589-7500(21)00174-6 PMC8545654

[91] Farah I, Stern D, Ramírez Y, et al. Food and beverage purchases at formal and informal outlets in Mexico. *Public Health Nutr*. 2023;26(5):1034–1034. 36285524 10.1017/S1368980022002324 PMC7614539

[92] Domínguez-Barreto AP, Farah I, López-Olmedo N, et al. Trends in food and beverage purchases in informal, mixed, and formal food outlets in Mexico: ENIGH 1994–2020. *Frontiers Public Health*. 2023;11:1151916–1151916. 10.3389/fpubh.2023.1151916 PMC10244666 37293617

[93] Colchero MA, Barrientos-Gutiérrez T. Guerrero-López CM, Bautista-Arredondo S. Density of alcohol-selling outlets and prices are associated with frequent binge drinking in Mexico. *Prev Med*. 2022;154:106921–106921. 34922993 10.1016/j.ypmed.2021.106921

[94] Prado-Galbarro FJ, Auchincloss AH, Pérez-Ferrer C, Sanchez-Franco S, Barrientos-Gutierrez T. Adolescent tobacco exposure in 31 latin american cities before and after the framework convention for tobacco control. *Int J Environ Res Public Health*. 2020;17(20):1–15. 10.3390/ijerph17207423 PMC7601699 33053821

[95] Prado-Galbarro FJ, Pérez-Ferrer C, Ortigoza A, et al. Early childhood development and urban environment in Mexico. *PLoS ONE*. 2021;16(11):1–14. 10.1371/journal.pone.0259946 PMC8598011 34788324

[96] Vaz C, Andrade AC, Silva U, et al. Physical disorders and poor self-rated health in adults living in four latin American cities: a multilevel approach. *Int J Environ Res Public Health*. 2020;17(23):1–12. 10.3390/ijerph17238956 PMC7730272 33276424

[97] Paiva ASS, Santos GF, Castro CP, et al. A scaling investigation of urban form features in Latin America cities. *PLoS ONE*. 2023;18(12):e0293518. 38109440 10.1371/journal.pone.0293518 PMC10727436

[98] Wang X, Rodríguez DA, Mahendra A. Support for market-based and command-and-control congestion relief policies in Latin American cities: effects of mobility, environmental health, and city-level factors. *Trans Res Part Policy Pract*. 2020;2021(146):91–108. 10.1016/j.tra.2020.12.004 PMC7611337 34295022

[99] Huertas JA, Palacio A, Botero M, et al. Level of traffic stress-based classification: A clustering approach for Bogotá, Colombia. *Transp Research Part D Transp Environ*. 2020;85:102420. 10.1016/j.trd.2020.102420 PMC7437968 32831580

[100] Higuera-Mendieta D, Uriza PA, Cabrales SA, Medaglia AL, Guzman LA, Sarmiento OL. Is the built-environment at origin, on route, and at destination associated with bicycle commuting? A gender-informed approach. *J Transp Geogr*. 2021;94:103120. 10.1016/j.jtrangeo.2021.103120 PMC8283281 34305337

[101] Mejia-Arbelaez C, Sarmiento OL, Vega RM, et al. Social inclusion and physical activity in ciclovía recreativa programs in Latin America. *Int J Environ Res Public Health*. 2021;18(2):1–24. 10.3390/ijerph18020655 PMC7828741 33466637

[102] Slovic AD, Indvik K, Soriano Martins L, et al. Climate hazards in Latin American cities: Understanding the role of the social and built Environments and barriers to adaptation action. *Clim Risk Management*. 2024;45:100625. 10.1016/j.crm.2024.100625 PMC11406151 39296795

[103] Santos GFd, Vives Vergara A, Fuentes-Alburquenque M, et al. Socioeconomic Urban Environment in Latin America: Towards a Typology of Cities. *Sustainability*. 2023;158(8):6380–6380. 10.3390/su15086380 PMC7617124 39898959

[104] Bakhtsiyarava M, Schinasi LH, Sánchez BN, et al. 2023 Modification of temperature-related human mortality by area-level socioeconomic and demographic characteristics in Latin American cities. *Soc Sci Med*. 1982;1(2023):317. 10.1016/j.socscimed.2022.115526 PMC9870751 36476939

[105] López-Olmedo N, Diez-Roux AV, Pérez-Ferrer C, et al. Climate Trends and Consumption of Foods and Beverages by Processing Level in Mexican Cities. *Front Nutr*. 2021;8:1–11. 10.3389/fnut.2021.647497 PMC8334732 34368204

[106] Carnalla M, Lopez-Olmedo N, Ramirez-Toscano Y, et al. Binge drinking associated with mean temperature: a cross-sectional study among Mexican adults living in cities. *Global Health*. 2024;20(1):29. 38609988 10.1186/s12992-024-01033-z PMC11010420

[107] Canto-Osorio F, Langellier BA, Unar-Munguia M, et al. Trends in the contribution of greenhouse gas emissions from food and beverage purchases in Mexico: 1989–2020. *Nutr J*. 2024;23(1):55. 38762743 10.1186/s12937-024-00955-z PMC11102158

