# Supplementary material for: Urban Environments, Health, and Environmental Sustainability: Findings From the SALURBAL Study
Source: J Urban Health. 2024 Nov 25;101(6):1087–103. doi: 10.1007/s11524-024-00932-1 (PMC11652555; doi:10.1007/s11524-024-00932-1)
Supplement: Supplementary file 1 — Supplementary file1 (DOCX 113 KB) [file 11524_2024_932_MOESM1_ESM.docx]

| **Supplement Table 1. Summary of research questions and key findings for SALURBAL Aim 1** | | |
| --- | --- | --- |
| **Categories of research questions** | **Area of focus** | **Select key findings** |
| **Differences across cities in life expectancy (LE) and mortality** | Differences in LE, and proportionate mortality across cities | Life expectancy (LE) and proportionate mortality by cause are highly heterogeneous across Latin American cities and are linked to area SES .[6]   - City LE varies across countries but also significantly within countries (within country differences as large as 7-10 years). - Cause-specific mortality also varied across cities, with some causes of death (unintentional and violent injuries and deaths) showing large variation within countries, whereas other causes of death (communicable, maternal, neonatal, and nutritional, cancer, cardiovascular disease and other noncommunicable diseases) varied substantially between countries. - Better city levels of education, water access, sanitation, and less overcrowding are associated with higher city life expectancy and a relatively lower proportion of communicable, maternal, prenatal, and nutritional conditions, and higher proportion of cancer and cardiovascular diseases and other non-communicable diseases deaths. - Denser cities had a higher proportionate mortality by violent injuries and less-fragmented and more-connected cities had a higher proportionate mortality by communicable, maternal and neonatal mortality. - Larger cities had shorter LE for men and had a relatively higher proportionate mortality by violence compared with smaller cities. |
|  | Differences in all cause and cause-specific mortality across cities | City size has no unique relation to mortality.[25]   - Latin American cities had similar all-cause mortality across different city sizes. However, the relationship of city size with mortality differed by cause. - Sexually transmitted infections, HIV/AIDs and homicides showed higher rates in larger cities (superlinear scaling), although the superlinear scaling of homicides was mostly driven by cities in Brazil. - Tuberculosis mortality was lower in larger cities in Mexico (sublinear scaling) but higher in larger cities in all other Latin American cities. - Deaths from other communicable, maternal, neonatal, and nutritional conditions, were linear or superlinear in Latin America. - Non-communicable diseases were mostly linear or superlinear (for some cancers and a few other NCDs (but not CVD or respiratory diseases which were mostly linear) in Latin America.   Road traffic mortality also varies substantially across cities.[28]   - It is lower in cities with higher population density, higher street connectivity, better social environment (a composite of access to water and sanitation, crowding and education) and those with a mass transit system such as a subway or BRT. - Cities with higher GDP per capita also had lower road traffic mortality. - In contrast, cities with more isolated built up areas had significantly higher road traffic mortality.   Homicide mortality also varies substantially across cities. [29, 71], [72]   - Youth and young adult homicides vary substantially across SALURBAL cities and sub-cities even after accounting for country. Higher sub-city education, higher GDP, and lower Gini were associated with lower homicide rates among men and women; higher isolation was associated with higher homicide rates. Larger cities and more dense cities also tended to have more homicides especially in men though associations were weakened after adjustment.[29] - In Brazilian cities higher income segregation, higher GINI index, higher GDP, lower unemployment, and lower education are all independently associated with higher homicide mortality.[71] - Homicides of women also vary substantially across cities. While findings suggest a slight decline in femicide rates over time between 2000 and 2019 the trends diverge in different countries, suggesting increasing rates in some countries like Mexico. Age specific trends suggest the persistence of higher rates among women 15-29 and 30-44 years of age over time. [72]   Overdose mortality rates also vary across cities. [73]   - In Mexico, fatal overdose rates were almost twice as high in urban compared to rural areas. However they also varied across cities, ranging from a high of 9.84 overdose deaths per 100,000 population over the period 2005-2021 in San Luis Rio Colorado (Sonora, northern Mexico) to 0.05 in San Juan Bautista Tuxtepec (Oaxaca, southern Mexico). Cities with the eight highest fatal overdose rates in the period were all on the US-Mexico border.[73]   City health care total amenable mortality varies more between than within countries, but city preventable amenable mortality varies more within than between countries. [26]   - Higher city SES and higher 5 year city growth are associated with lower total amenable mortality. - Higher fragmentation is associated with lower amenable mortality in smaller cities but with higher amenable mortality in larger cities. - Larger population size was associated with higher amenable mortality due to preventable conditions regardless of fragmentation.   In Chile, cities in with higher residential overcrowding had higher excess mortality from COVID -19[31] and in Brazil cities with higher income inequality and higher income segregation experienced higher COVID-19 mortality rates.[30] |
|  | Differences in infant mortality across cities | Infant mortality rates (IMR) vary substantially across cities within a country. Sometimes within country differences are as large or larger than average between country differences.[27]   - - Better living conditions, service provision, and public transportation are associated with lower IMR in cities.[27]   - Cities where women are more empowered tend to have lower infant mortality rates.[33]   Greater political representation of women is associated with better infant and child survival in Brazil. [34] |
|  | Changes over time in city mortality | Economic fluctuations are linked with mortality.[35] Increases in GDP per capita are associated with increases in total mortality and in mortality due to CVD, cancer, diabetes, respiratory infections, and road traffic injuries but with decreases in homicide rates.  A 10 μg/m^3^ increase in monthly PM_2.5_ was associated with an increase of 1.3 % (95 % confidence interval [CI], 0.4 to 2.2) in cardiovascular mortality and a 0.9 % increase (95 % CI -0.6 to 2.4) in respiratory mortality. Increases in mortality risk associated with higher PM2.5 were larger in men and became larger as age increased.[36] |
| **Differences within cities in LE and mortality** | Differences in LE within cities | LE varies significantly within cities and is linked to area SES.   - Differences across sub-city units are as large as 10-17 years in some cities.[22] - Areas of cities with higher socioeconomic status have longer life expectancy than areas with lower SES. Difference between 90^th^ and the 10^th^ percentile of area education were 8-11.8 years in Santiago and Panama City, 3.5-5.3 years in Buenos Aires and Belo Horizonte, 2.3-2.9 in Mexico City and 0.6-0.7 years in San Jose with associations consistently stronger in women than in men.[22] - This pattern is also observed for smaller areas (neighborhoods) within cities. In both Cordoba[8] and Argentina[24] LE varies significantly across small areas: 3.2 and 4.6 years in women and men in Cordoba comparing 90^th^ to 10^th^ percentile[8] and 2.9 years and 5.3 years among women and men in CABA comparing 90^th^ to 10^th^ percentile.[24] - Analyses of smaller areas within cities also shows associations of higher area SES with longer life expectancy: 3 years and 2.6 years for highest vs lowest decile of area education in men and women in Cordoba[8] and 5.6 years and 3 years for men and women in CABA.[24] |
|  | Differences in cause specific mortality within cities | - In 28 large cities higher city greenness was associated with smaller subcity* education disparities in violence-related mortality (in men only) but larger subcity education disparities in LE and CVD mortality. [74] - Steep terrain, nighttime travel, the absence of dedicated infrastructure and collisions with large vehicles are associated with more cyclist mortality due to road crashes in Bogotá.[75] - Within cities COVID-19 mortality and excess mortality during the pandemic was higher in areas of lower SES than in areas of higher SES[31, 32] and small area inequities in mortality within cities increased during the pandemic.[76] |
| **Differences across and within cities in birth rates and perinatal outcomes** | Differences in adolescent birth rates across cities and areas within cities | Adolescent birth rates (ABR) vary significantly across cities within countries.[77]   - - Cities with higher homicide rates and larger population growth tend to have higher ABR.   - Better sub-city living conditions and higher education are associated with lower ABR.   Adolescent birth rates are higher in cities with more gender inequality, where women receive fewer years of education, and where women have less access to routine preventive healthcare. Cities that had at least one woman mayor also tended to have lower ABR than those who did not. [78]  In Mexico, subcity areas (municipalities) with higher density of education, recreational, and health care facilities had lower ABR whereas municipalities with higher density of on-premises alcohol outlets have higher ABR. Increases over time in density of recreational facilities, pharmacies, and off premise alcohol outlets are associated with reductions in ABR.[79] |
|  | Differences in birthweight and C sections across cities and areas within cities | There is wide variability in the rate of cesarean sections across cities. Overall, 52% of analyzed births were via cesarean section, with large differences between cities (range 13-91%). A lower rate was observed for mothers with lower education-level compared to higher (39% vs 61%). Higher subcity area educational attainment and higher city GDP were associated with higher cesarean rates.[80]  Of the total low birth weight (LBW) variability across sub-city units, most is between countries but 8% is between cities within a country, and 16% was within cities. Low maternal education was associated with higher prevalence of LBW. In contrast, higher subcity area education and higher city social environment were independently associated with higher LBW prevalence. Cities with larger populations had higher prevalence of LBW.[81]  The implementation of a smoking ban law did not cause a significant impact on low birth weight rates in Chile in the 2.5 years following the law’s implementation compared to the 2 years prior to implementation. However, smoking prevalence decreased significantly in women in cities after the law’s implementation. [82] |
| **Inequities in health risks factors, mental health and self- reported health** | Inequities linked to individual and household level SES indicators | Lower education level is associated with more diabetes among women. In men this association is weaker and varies by country and city socioeconomic development with inverse associations emerging as city socioeconomic development (an index including water access, sanitation, overcrowding and education) increases.[37]  Lower education level is associated with more obesity among women. In men the association varies depending on city socioeconomic development with inverse associations emerging in cities with better socioeconomic development.[38]  Higher individual-level education was associated with higher odds of hypertension in men and lower odds in women. In men an inverse association began to emerge as subcity area education SES increased. Analyses of measured blood pressure in a subsample showed an inverse association in men, suggesting that differences in access to health care (and diagnosis of hypertension) play a role in social patterning.[39]  In 11 cities, individuals employed in informal jobs had a 27% higher prevalence of major depressive symptoms (Prevalence Ratio [PR]: 1.27; 95% Confidence Interval [CI]: 1.00, 1.62) compared to those in formal jobs. The prevalence of depressive symptoms among individuals with informal jobs was higher compared to those with formal jobs in both women (PR: 1.36, 95% CI: 1.06, 1.74) and men (PR: 1.22; 95% CI: 0.90, 1.65) after adjustment for age education and household characteristics .[83]  In 11 cities after adjusting for respondent age, gender, and city, we found a higher risk of diarrhea associated with higher household deprivation scores. Specifically, we found that the odds of diarrhea for children living in a mild and severe deprived household were 1.04 (95% CI 0.84-1.28) and 3.19 times (95% CI 1.80-5.63) higher, respectively, in comparison to households with no deprivation. [84]  Country-specific analyses:   - In Brazil, after adjusting for age, gender, and education, Black people had nearly 20% higher prevalence of fair/poor SRH than did White people, and Brown people had more than 10% higher prevalence of fair/poor SRH than did White people. We also found that racial inequities in SRH were larger in more segregated than less segregated cities, for both income and race segregation: the more segregated the cities, the greater the racial disparity in fair/poor SRH. [41] - In Argentina higher education was associated with less diabetes, hypertension, obesity, smoking and binge drinking with the exception of diabetes in men and binge drinking and smoking in women. Results also suggested that city education may modify associations of individual and neighborhood education with health risk factors. [85] |
|  | Inequities linked to area-based socioeconomic (SES) indicators | Several SALURBAL papers explored the associations of city and neighborhood SES with NCD risks factors after controlling for individual-level characteristics. Findings were mixed with some studies finding no associations (eg city SES was unrelated to prevalence of diabetes, hypertension or obesity [37-39]), but others finding positive associations (eg higher subcity unit SES was related to higher odds of hypertension).[39] Findings in children were also mixed: higher subcity education was associated with lower odds of child obesity but better subcity living conditions (as indexed by households with piped water inside the dwelling; overcrowding; and population aged 15-17 attending school) was associated with higher odds of obesity.[86]  Higher city labor women's empowerment was associated with a lower prevalence of overweight/obesity in women. Similar albeit weaker associations were observed in men. Associations were stronger at higher levels of individual-level education and in subcity areas with better living conditions. Unexpectedly even after adjusting for country GDP higher city Gini was associated with lower prevalence of obesity/overweight in men.[87]  In country specific analyses in Argentina higher neighborhood education was associated with lower odds of obesity in both genders, with lower odds of diabetes and hypertension in women, and with higher odds of binge drinking in women. Higher city education was associated with higher odds of diabetes among women.[85] |
| **Differences across cities and subcities in health risk factors, mental health, and self-reported health** | Health risk factors and infectious disease incidence | Urban form, green space and transportation environments   - Living in subcity areas with higher intersection density is associated with higher body mass index, obesity, and diabetes. Living in more fragmented cities is associated with lower BMI and obesity. Living in greener subcity areas is associated with lower BMI, obesity, and diabetes. Higher subcity population density is associated with less diabetes. [47] - Higher city fragmentation and presence of mass transit are related to higher prevalence of hypertension. Higher sub-city population density is related to lower prevalence of hypertension. Higher sub-city intersection density was associated with higher odds of having hypertension. Except for intersection density and pop density (which remains borderline sig), associations were attenuated after adjustment for country. An inverse association of greenness with continuous blood pressure emerged after country adjustment. [48] - Across 159 large cities in six Latin American countries cities, 8% of children aged 1-5 years were overweight or obese, but prevalence varies substantially across cities ranging from 4% to 25%. Higher isolation of urban patches was associated with lower rates of excess weight among children.[86] - Longer travel times and delay times are associated with lower vegetable consumption. Longer travel delays are also associated with higher odds of moderate or frequent consumption of sugar-sweetened beverages. Associations were stronger in larger cities[53] but there was no association between city-level travel time during peak traffic hours and odds of obesity or diabetes.[54] - In 11 cities, greater perceived park proximity was associated with increased odds of park use. Additional factors that were positively associated with park use were neighborhood formality and built environment characteristics, including paved streets and sidewalks.[88] - High greenness of vegetation is associated with lower incidence of dengue. This association is modified by socioeconomic vulnerability: while a positive association was observed in the least vulnerable census tracts, the association was negative in the most vulnerable areas.[89] - Lower sub-city weekly mobility was associated with lower incidence of COVID-19 the following week.[90]   Food environments   - In Mexico 15% of food and beverage purchases were in the formal sector (supermarket (10%) restaurants, cafes and bars (4%) and convenience stores (1%)), 14% were in the informal sector (street vendors (7%), street markets (4%) , and acquaintances (3%)) and 70% were in mixed outlets including small neighborhood stores (30%), specialty stores (25%), public markets (8%) and low budget restaurants (7%). Higher household income and higher urbanicity were associated with greater proportion of expenditures in the formal sector and lower expenditures in the informal sector. Small neighborhood stores and specialty stores are the main source of food and beverage purchases in Mexico, across all income strata and levels of urbanicity.[91] Over time we observed an increase in food purchases from the formal sector, nonetheless, the mixed sector remains the predominant food source in Mexico, especially small-neighborhood stores. [92] - In Mexico, in urban and rural areas combined children aged 5-19 years living in areas with increasing density of fruit and vegetable stores have declines (or slower increases) in BMI during childhood and adolescence. Increases in the density of convenience stores and supermarkets was associated with increases in BMI. When analyses were restricted to urban areas (ie SALURBAL cities) there was an inverse association between increases in specialty food stores and declines (or slower increases) in BMI (β= -0.789 kg/m^2^ in third quartile, and β= -1.204 kg/m^2^ in fourth quartile). However other associations were not statistically significant.[50] - In 53 SALURBAL cities in Mexico, adults living in neighborhoods that saw a decline in fruit and vegetable store density and a simultaneous increase in chain convenience store density between 2010 and 2016 experienced higher incidence of diabetes between 2010 and 2016, compared to adults who lived in neighborhoods where fruit and vegetable and convenience stores did not change. [51] - In 53 SALURBAL cities in Mexico declines in neighborhood-level density of fruit and vegetable stores between 2010 and 2016 were associated with higher levels of blood pressure in 2016 but the association was attenuated after controlling for individual-level and area-level covariates. In addition, a prior increase in density of large supermarkets was associated with higher blood among adults with undiagnosed hypertension. [52] - In Mexico, individuals living in urban areas with high density of alcohol-selling outlets and low alcohol prices have higher prevalence of weekly binge drinking.[93]   Legal environments   - Using data from 132,065 students in 31 cities we found that ratification of the 2003 Framework Convention on Tobacco Control was associated with reductions in adolescent reports of tobacco use and exposure to smoking at home, with more tobacco education, and with more retailer refusals to sell them cigarettes. However there was little change in reported exposure to secondhand smoke exposure outside the home and no change in exposure to tobacco media/promotions, suggesting that policies related to secondhand smoke and advertising need strengthening. There was wide variation in adolescent exposure to tobacco between cities within countries, which suggested major heterogeneity of policy implementation at the local level.[94] |
|  | Mental health and development | Longer commuting, experience of traffic delays, commuting by personal vehicle, and worse access to transit are associated with more depressive symptoms.[46]  In Mexico (17258 respondents in 84 cities) the amount of greenness measured by neighborhood NDVI was associated with lower odds of depressive symptoms. There was some evidence that, when neighborhood-level greenness is accounted for, the broader availability of greenspace outside of the neighborhood may be associated with smaller odds of depressive symptoms. We found no statistically significant associations for % greenspace in a neighborhood or urban parks.[45]  In Mexico, the presence of more libraries and day care centers in cities and sub-cities is associated with better early childhood development. [95] |
|  | Self-reported health | Across 112 cities in 4 countries (Argentina , Brazil Chile, Colombia), better services at the subcity level (specifically a services score indicating access to water and sanitation) and at the city-level (a summary score of services, crowding and education) were associated with lower odds of poor self reported health. (OR = 0.93 per SD, 95% CI = 0.87-0.99 and OR = 0.90 per SD, 95% CI = 0.82-0.99 respectively). [43] High subcity physical disorder was related to worse self-rated health in 4 cities.[96]  In both genders, both lower levels of city social environment index (SEI ) and lower GDP per-capita were associated with poor SRH, even after adjusting for each other, with somewhat stronger associations for SEI as compared to GDP per capita. In addition, we found that GPD per-capita modified the associations between age and poor SRH, such that in middle-aged adults the age-SRH associations were stronger in cities with higher GPD per-capita. This interaction was less clear for city-SEI.[44] |
| **Health-relevant urban environments: heterogeneity and predictors** | Urban form, street design and transportation | Urban form and street design features   - Urban built environments and street designs in Latin America are heterogeneous but can be clustered into distinct typologies based on landscape or street design.[55] - Using data from 208 cities we investigated whether certain city landscape profiles were more likely to show positive or negative health and environmental co-benefits. Overall, 27% of cities fell into the positive co-benefits group, whereas 44% fell into the negative co-benefits group. Cities with the scattered pixels profile (representing low fragmentation, high isolation, and more compact development) were more likely than other city profiles to have positive co-benefits. In contrast, the contiguous large inkblot cities (higher fragmentation and complex shape, and often very large) were the least likely to be in the positive co-benefits class.[56] - Many built environment features (total urbanized area, number of of urban patches, and number of streets and intersections) scale sublinearly indicating a lower value than would be expected if they increased proportionally with population size.[97]   Car use   - Cities in Latin America are experiencing increased motorization over time. Development fragmentation, urban form complexity and circuity of the street network are associated with increases in motorization. In addition, the increase in motorization rates between 2010 and 2015 showed a negative association with population density. [61] - Policy approaches to reduce car traffic congestion are more acceptable among those who experience traffic delays, have a child with respiratory illness, experience higher levels of air pollution, who live in cities with lower population levels, and who have had prior experience with traffic bans.[98]   Cycling infrastructure and active transportation:   - One third of all trips in five large Latin American cities are walking-only and walking for transportation may be underestimated as incidental walking to other modes are not accounted for. Women in these cities walk more than men, and children walk more than adults.[62] - Those with higher SES have lower odds of using bicycles. Odds of bicycle use have increased over time, especially among high SES populations.[56] - A segment level measure of traffic stress is associated with the number of bicyclists’ fatal and non-fatal collisions in Bogotá. [99] - In Bogota, although both women and men increased the standardized number of bicycle commuters, male commuters show a steeper trend than women, evidencing the widening gender gap in bicycle commuting over time. Bicycle commuting was negatively associated with household motor vehicle ownership, steeper terrain slope, longer commute distance, and scarce low-stress roads at trip origin and route. [100] - Ciclovías are a socially inclusive program to support walking, cycling and physical activity which provides a space that facilitates physical proximity, exposure to new communities and environments, and relationships between different socioeconomic groups.[101] |
|  | Greenness and green space | Greenness and green space:   - SALURBAL produced the first urban green space (UGS) map for Latin America, which enables studies to measure area, spatial configuration, and human exposures to UGS, facilitating studies on the relationship between UGS and human exposures to environmental hazards, public health outcomes, and environmental justice issues in Latin American cities.[57] - Most SALURBAL cities and sub-cities experienced increases in greenness over time. Cities and sub-cities with higher overall SES tend to be less green. Cities with higher SES are experiencing higher increases in greenness over time; however, the opposite was observed for sub-cities. The findings challenge the belief that places with higher SES have better access to environmental resources and amenities; instead, this relationship is context-dependent.[59] - In SALURBAL cities better economic conditions were associated with lower baseline greenness in 2011, which contributed to faster warming over time. There was modest evidence that this faster warming associated with lower baseline greenness and improved economic conditions was partially offset by cooling from recent greening (2001–2022) in cities of better economic conditions. This offset was more evident in arid cities.[60] |
|  | Air pollution, temperature and other climate hazards | In 2015 nearly 60% of the population of Latin American cities lived in areas with air pollution levels above the then defined WHO-AQG of 10 μg/m3 annual average.[64]  Larger cities, cities with higher GDP, higher motorization, higher congestion tended to have higher PM2.5. In contrast cities with higher population density had lower levels of PM2.5. In addition, at the sub-city level, higher intersection density was associated with higher PM2.5 and more green space was associated with lower PM2.5. [64]  Of the roughly 236 million urban residents in 236 SALURBAL cities observed, 85% lived in neighborhoods with ambient annual NO2 above WHO guidelines. Higher neighborhood-level educational attainment, closer proximity to the city center, and lower neighborhood-level greenness were associated with higher ambient NO2. At the city level, higher vehicle congestion, population size, and population density were associated with higher ambient NO2.[65]  In a survey of 126 cities, city representatives reported a median of three climate hazards. The most reported hazards were storms (61%) water scarcity (57%) extreme temperature (52%) and wildfires (51%). Thirty-eight percent of cities reported four or more distinct types of hazards. Although most cities reported taking actions to reduce vulnerability to climate change, 23% reported no actions at all. The most frequently reported actions were hazard mapping and modeling (47%) and increasing vegetation or greenspace coverage (45%). The most frequently reported challenges in addressing climate hazards were urban environment and development (43%) and living conditions (35%). Access to data, migration, public health, and safety/security were rarely reported as challenges. Climate hazards are recognized, but adaptation responses are limited and that many important challenges to response action are not fully understood.[102] |
|  | Social and economic features | Census data from 371 urban agglomerations were used to identify socioeconomic typologies of cities. Five socioeconomic clusters patterned by country were identified. The main features of each cluster were: low-education cities in Northeast Brazil; low-unemployment cities in Peru and Panama; high-education cities in Argentina, Chile, Colombia, Costa Rica, Nicaragua and Mexico; high female labor participation, with high primary education in Argentina and low primary education in Brazil; and low female labor participation and low education in Brazil, Colombia, El Salvador, Guatemala, and Mexico.[103] |
| **Impacts of climate change on health in cities** | Heat and mortality | A substantial proportion of deaths are attributable to non-optimal ambient temperatures. The proportion of deaths attributable to temperatures below the optimal temperature is 5.1% and the percent attributable to temperatures above the optimal temperature is 0.7%. At very high temperatures small increases in temperatures are associated with high increase in risk (higher than those observed for very low temperatures). Older populations are particularly vulnerable. Of the causes studied, respiratory and cardiovascular conditions are the most impacted by temperature. [7]  Cities in the highest compared to the lowest tertile of income inequality have all-age cold-related excess mortality that is, on average, 3.45 percentage points higher (95% CI: 0.33, 6.56). Higher poverty and higher segregation were also associated with higher cold EDF among those 65 and older. Large, densely populated cities, and cities with high levels of poverty and income inequality experience smaller heat EDFs compared to smaller and less densely populated cities, and cities with little poverty and income inequality.[104]  City greenness may buffer adverse heat impacts in arid cities. Among the 79 cities in arid climate zones those with moderate and high greenness had lower heat-EDFs compare to cities with lowest greenness. [67] |
|  | Heat and birth weight | Higher-than-average temperatures during the entire gestational period are associated with lower birthweight, particularly in Mexico and Brazil. The cumulative effect of temperature on birthweight is mostly driven by exposure to higher-than-average temperatures during the last months of gestation: we find that during months 7-9 of gestation the associations between higher temperature and lower birthweight are of greatest magnitude. Higher maternal education may attenuate the temperature-mortality associations. [66] |
|  | Climate and diet | In Mexico, lower precipitation and higher temperatures are associated with lower consumption of unprocessed foods and higher consumption of ultra-processed foods. [105]  Higher municipality mean annual temperature was associated with mire binge drinking in Mexico. [106]  Households with higher education levels and those in more urbanized areas contributed more to dietary GHGE across the full period. However, households with lower education levels and those in rural areas had the highest increase in these emissions from 1989 to 2020.[107] |
| * Subcities are administrative units within cities such as municipalities or similar. | | |
